# Surgeons’ Ability to Predict the Extent of Surgery Prior to Cytoreductive Surgery with Hyperthermic Intraperitoneal Chemotherapy

**DOI:** 10.1245/s10434-020-08237-8

**Published:** 2020-02-12

**Authors:** Judith E. K. R. Hentzen, Willemijn Y. van der Plas, Lukas B. Been, Frederik J. H. Hoogwater, Robert J. van Ginkel, Gooitzen M. van Dam, Patrick H. J. Hemmer, Schelto Kruijff

**Affiliations:** 1 Department of Surgery, Division of Surgical Oncology, University Medical Center Groningen, University of Groningen, Groningen, The Netherlands; 2 Department of Surgery, Division of Hepatopancreatobiliary Surgery and Liver Transplantation, University Medical Center Groningen, University of Groningen, Groningen, The Netherlands; 3 Department of Nuclear Medicine and Molecular Imaging, University Medical Center Groningen, University of Groningen, Groningen, The Netherlands

## Abstract

**Background:**

The extent of surgery (ES) during cytoreductive surgery with hyperthermic intraperitoneal chemotherapy (CRS + HIPEC) is a well-known risk factor for major postoperative morbidity. Interestingly, the reliability of surgeons to predict the ES prior to CRS + HIPEC is unknown.

**Methods:**

In this prospective, observational cohort study, five surgeons predicted the ES prior to surgery in all consecutive patients with peritoneal metastases (PM) who were scheduled for CRS + HIPEC between March 2018 and May 2019. After the preoperative work-up for CRS + HIPEC was completed, all surgeons independently predicted, for each individual patient, the resection or preservation of 22 different anatomical structures and the presence of a stoma post-HIPEC according to a standardized ES form. The actual ES during CRS + HIPEC was extracted from the surgical procedure report and compared with the predicted ES. Overall and individual positive (PPV) and negative predictive values (NPV) for each anatomical structure were calculated.

**Results:**

One hundred and thirty-one ES forms were collected from 32 patients who successfully underwent CRS + HIPEC. The number of resections was predicted correctly 24 times (18.3%), overestimated 57 times (43.5%), and underestimated 50 times (38.2%). Overall PPVs for the different anatomical structures ranged between 33.3 and 87.8%. Overall, NPVs ranged between 54.9 and 100%, and an NPV > 90% was observed for 12 anatomical structures.

**Conclusions:**

Experienced surgeons seem to be able to better predict the anatomical structures that remain in situ after CRS + HIPEC, rather than predict the resections that were necessary to achieve a complete cytoreduction.

**Electronic supplementary material:**

The online version of this article (10.1245/s10434-020-08237-8) contains supplementary material, which is available to authorized users.

Cytoreductive surgery combined with hyperthermic intraperitoneal chemotherapy (CRS + HIPEC) is used in highly selected patients with peritoneal metastases (PM) from gastrointestinal, gynecological, or primary peritoneal cancers.^1^–^4^ This treatment strategy combines the surgical removal of all macroscopically visible disease with perfusion of the abdominal cavity with heated chemotherapy to eradicate residual microscopic disease. Cumulative scientific evidence showed an important improvement in survival outcomes compared with systemic chemotherapy alone.^5^–^7^

CRS + HIPEC is accompanied by a high treatment-related mortality rate of 0–8% and grade 3–4 morbidity rate of 18–52% in experienced centers.^8^–^14^ In addition, CRS + HIPEC negatively impacts quality of life (QoL) up to one year after surgery.^15^,^16^ For clinicians and patients, it remains a challenge to weigh the potential survival benefit from CRS + HIPEC against the risk of substantial treatment-related morbidity, mortality, and potentially diminished QoL.

The extent of surgery (ES) has been identified as an independent risk factor for treatment-related morbidity and mortality.^17^–^20^ The ES during CRS obviously depends on the extent of involved organs because of metastases or the close relation with metastases, which makes it surgically mandatory to remove the affected anatomical structures. The extent of peritoneal disease widely varies between patients. To predict postoperative outcomes prior to surgery, it is pivotal for every surgeon to appreciate the ES before deciding, in a patient-shared decision, to proceed with CRS + HIPEC.

However, the correlation between the predicted ES by experienced surgeons in advance and the actual ES during CRS is unknown. As such, the aim of the present study was to determine the correlation between the predicted and actual ES during CRS.

## Methods

### Design, Setting, and Participants

In this prospective, observational cohort study, five surgeons from one Dutch tertiary referral center (University Medical Center Groningen) predicted the ES prior to surgery in all consecutive patients with histologically proven PM of any origin who were scheduled for CRS + HIPEC. Surgeons with extensive experience in gastrointestinal surgery and CRS + HIPEC procedures were asked to participate in this study. The learning curve from these surgeons has been studied before and was published by the Dutch Peritoneal Oncology Group (DPOG) elsewere.^21^ Data on patient and tumor characteristics, as well as operative and postoperative characteristics, were collected prospectively and stored in our institutional database in compliance with the Declaration of Helsinki.^22^ The Institutional Ethics Committee of the University Medical Center Groningen approved the study protocol (METc201800157). Written informed consent was obtained from all patients.

Every surgeon predicted the ES before each CRS + HIPEC procedure based on the information from our standardized preoperative work-up for CRS + HIPEC and after our weekly multidisciplinary oncology meeting, which are described later. Each surgeon predicted the ES for every patient undergoing CRS + HIPEC irrespective of being the operating surgeon of the case. The surgeons anonymously filled in the ‘Extent of Surgery’ form to ensure a standardized way of estimating the ES (Appendix 1). In summary, this ES form included 22 anatomical structures that might be resected during CRS + HIPEC and a separate question about the presence of a stoma post-HIPEC. The surgeons were instructed to predict the resection or preservation of each anatomical structure and to predict the presence or absence of a stoma post-HIPEC. In cases in which an open–close procedure (i.e. non-therapeutic laparotomy) was expected by the surgeon, he indicated this on the ES form and did not make any predictions regarding the anatomical structures or the presence of a stoma.

After surgery, the ES forms were compared with the actual ES during CRS + HIPEC retrieved from the surgical procedure report. For every anatomical structure, four scenarios could occur: (1) the surgeon predicted correctly that an anatomical structure would be resected during CRS + HIPEC; (2) the surgeon predicted correctly that an anatomical structure would remain in situ after CRS + HIPEC; (3) the surgeon predicted incorrectly that an anatomical structure would be resected during CRS + HIPEC; or (4) the surgeon predicted incorrectly that an anatomical structure would remain in situ after CRS + HIPEC. Because our study aimed to correlate the predicted ES with the actual ES, only fully completed ES forms from patients who underwent complete CRS + HIPEC were included in the final analyses.

### Primary and Secondary Outcomes

The primary outcome was the overall ability of the surgeons to predict the ES prior to CRS + HIPEC. The primary outcome was divided into overall positive (PPVs) and negative predictive values (NPVs) per anatomical structure. A high PPV suggests that the surgeon is well able to predict if the anatomical structure will be resected, whereas a high NPV indicates that the surgeon is well able to predict if the anatomical structure will remain in situ. Secondary outcomes included overall and individual PPVs and NPVs for the presence of a stoma post-HIPEC, individual PPVs and NPVs per anatomical structure, overall sensitivity and specificity per anatomical structure, and the occurrence of major postoperative complications. Major postoperative complications were defined as grade III or higher according to the Clavien–Dindo classification system.^23^

### Preoperative Evaluation and Staging for Cytoreductive Surgery (CRS) + Hyperthermic Intraperitoneal Chemotherapy (HIPEC)

All referred patients with PM underwent a standardized preoperative work-up for CRS + HIPEC consisting of a clinical examination, laboratory testing, and a thoracic, abdominal, and pelvic computed tomography (CT) scan to quantify the extent and resectability of peritoneal disease and to rule out other distant metastases. If deemed necessary, additional magnetic resonance imaging (MRI) was performed to further investigate the extent of PM. In patients with colorectal PM, diagnostic laparoscopy (DLS) was routinely performed by one of our HIPEC surgeons to pathologically confirm the presence of PM and to systematically assess the extent and resectability of peritoneal disease according to the peritoneal cancer index (i.e. DLS PCI).^24^

Afterwards, during a weekly multidisciplinary oncology meeting, results from all patients were discussed and eligibility for CRS + HIPEC was determined. In general, patients who were candidates for CRS + HIPEC had limited and resectable PM with no evidence of extra-abdominal disease and were deemed fit for extensive abdominal surgery. Up to three resectable liver metastases were not considered as an absolute contraindication. Extensive small bowel resection resulting in short bowel syndrome was an absolute contraindication. No definite PCI limitations were used in patients with PM from pseudomyxoma peritonei (PMP), low-grade appendiceal mucinous neoplasm (LAMN), or mesothelioma. The absolute PCI cut-off point to perform CRS + HIPEC in patients with colorectal PM was 20.

### CRS with HIPEC

Every CRS + HIPEC procedure was performed by two HIPEC surgeons according to our standardized Dutch HIPEC protocol.^25^ CRS was performed only in patients whereby both surgeons judged the PM as completely resectable. In addition, HIPEC was performed only after a complete cytoreduction was achieved.

Each procedure started with an exploratory laparotomy to assess the extent of peritoneal disease in an open setting (i.e. laparotomy PCI). In cases of extensive or non-resectable PM, the procedure was prematurely terminated (i.e. open–close procedure). When the patient was deemed eligible for CRS + HIPEC, all macroscopically visible disease was removed by performing peritonectomies and organ resections. The completeness of cytoreduction (CC) score was determined at the end of the cytoreduction.^24^ The cytoreduction was considered complete if a CC score of 0 or 1 was established (i.e. no residual tumor or residual tumor deposits < 2.5 mm, respectively).

When complete cytoreduction was achieved, HIPEC was performed for 90 min at a temperature of 41–42 °C by using the open Coliseum technique. Mitomycin C (35 mg/m^2^) was used in patients with colorectal PM, LAMN, PMP, and small bowel carcinoma. A combination of cisplatin (50 mg/m^2^) with doxorubicin (15 mg/m^2^) was used in patients with mesothelioma.

Hereafter, the fluid was evacuated from the abdominal cavity and reconstruction surgery including bowel anastomoses with or without a stoma was performed. Patients were admitted to the intensive care unit for strict monitoring for at least one day, and were transferred to the nursing ward when cardiac and pulmonary functions were stable.

### Data Collection

Relevant data on patient characteristics, tumor characteristics, operative characteristics, and postoperative morbidity and mortality were extracted from a merged prospectively maintained institutional database. Postoperative complications within 90 days after surgery were registered according to the Clavien–Dindo classification system.^23^ Operative details including the anatomical resections, the presence of a stoma post-HIPEC, the operation time, and total blood loss were retrospectively extracted from the surgical procedure reports.

All fully completed ES forms from patients who underwent complete CRS + HIPEC were registered per surgeon and compared with the actual ES. For every anatomical structure, the four previously described possible scenarios were identified and registered.

### Statistical Analyses

Categorical variables are reported as number (*n*) and percentages (%), and continuous variables are reported as median (interquartile range [IQR]). The PPV, NPV, sensitivity, and specificity for each anatomical structure and the presence of a stoma post-HIPEC were calculated in total and per surgeon. In addition, the prevalence of all resections during CRS + HIPEC was determined according to all ES forms. For example, when only one patient underwent a stomach resection during CRS + HIPEC, the total prevalence to correctly predict a stomach resection in this study cohort was five, as five surgeons filled in an ES form.

Thereafter, three categories were created to further classify the surgeon’s ability to correctly predict the resection or preservation of each anatomical structure. The three categories consisted of (1) good PPV/NPV (i.e. > 80%); (2) moderate PPV/NPV (i.e. 50–80%); and (3) poor PPV/NPV (i.e. < 50%). The prevalence of each anatomical resection was taken into account when these categories were interpreted. All statistical analyses were conducted using SPSS Statistics version 24.0 (IBM Corporation, Armonk, NY, USA).

## Results

**Fig. 1:**
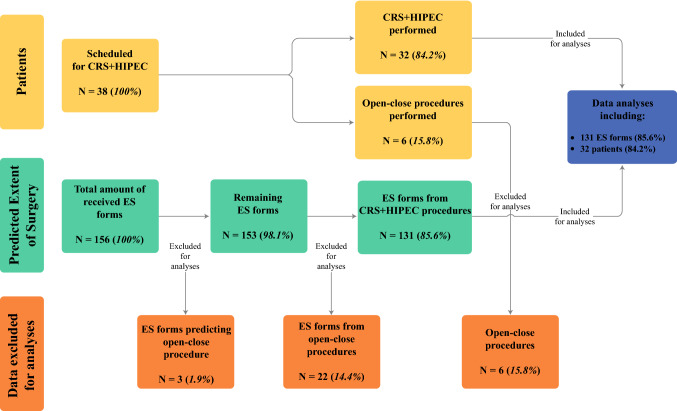
Overview of the selection process of collected ES forms for final analyses, *CRS* cytoreductive surgery, *HIPEC* hyperthermic intraperitoneal chemotherapy, *ES* extent of surgery

Thirty-eight consecutive patients underwent an exploratory laparotomy for potential CRS + HIPEC in our academic center between March 2018 and May 2019; 156 ES forms were completed during this study period. Because of the aim of the study, not all completed ES forms could be used for final analyses. Figure 1 provides a structured flow chart explaining this selection process. In six patients (15.8%), it was not possible to correlate the corresponding 24 ES forms (15.4%) with the actual ES, as an open–close procedure occurred. Interestingly, in only 2 of these 24 ES forms (8.3%) was an open–close procedure correctly predicted in advance. In addition, 1 of the remaining 132 ES forms was also excluded for analyses as the surgeon incorrectly predicted an open–close procedure and therefore did not make any predictions about the resection of the different anatomical structures. In summary, the data presented in this manuscript are based on 131 ES forms from 32 patients who successfully underwent CRS + HIPEC.

### Patient and Tumor Characteristics

Table 1 provides an overview of the baseline characteristics. The majority of patients had already undergone abdominal surgery in the past (90.6%) and were diagnosed with a metachronous onset of PM (56.3%) The most commonly treated tumor types were colorectal (77.4%) and appendiceal (12.9%) cancer. In all patients, radiological examinations were performed during the preoperative evaluation for CRS + HIPEC to assess the extent of disease and included CT for 32 patients (100%), MRI for 10 patients (31.3%), and positron emission tomography (PET)/CT for 12 patients (37.5%). Median time between CT and CRS + HIPEC was 4 weeks (IQR 1–6) and median time between MRI and CRS + HIPEC was 3 weeks (IQR 1–6). Furthermore, half of the patients underwent DLS in our center to pathologically confirm the presence of PM and to systematically assess the extent and resectability of peritoneal disease according to the PCI. DLS was refrained in the other half of patients because a DLS or exploratory laparotomy was recently performed to confirm the presence of PM and to investigate the extent of peritoneal disease.

**Table 1:** Baseline characteristics from all 32 patients who successfully underwent CRS + HIPEC

Patient characteristics	
Age, years (median [IQR])	59 [53–71]
Sex, male	13 (40.6)
BMI, kg/m^2^ (median [IQR])	24.9 [21.7–28.1]
ASA score	
1	2 (6.3)
2	29 (90.6)
3	1 (3.1)
Prior CRC surgery	29 (90.6)
Prior chemotherapy	10 (31.3)
*Tumor characteristics*	
Primary tumor	
Appendix	5 (15.6)
Right colon	7 (21.9)
Transverse colon	1 (3.1)
Left colon	3 (9.4)
Sigmoid	8 (25.0)
Rectum	6 (18.8)
Small bowel	1 (3.1)
Signet cell histology	5 (15.6)
T stage of the primary tumor	
≤ 3	9 (34.6)
4	17 (65.4)
*N* stage of the primary tumor	
0	6 (24.0)
1	9 (36.0)
2	10 (40.0)
M stage of the primary tumor	
0	15 (55.6)
1	12 (44.4)
Onset of PM	
Synchronous	14 (43.8)
Metachronous	18 (56.3)
Synchronous liver metastases	2 (6.3)
Primary tumor in situ	6 (18.8)
Presence of a stoma pre-HIPEC	6 (18.8)
Preoperative evaluation for CRS + HIPEC	
HIPEC indication	
Colorectal PM	24 (77.4)
PMP	2 (6.5)
LAMN	4 (12.9)
Mesothelioma	1 (3.2)
Small bowel carcinoma	1 (3.2)
Preoperative imaging	
CT scan	32 (100.0)
MRI scan	10 (31.3)
PET scan	12 (37.5)
DLS routinely performed, yes	16 (50.0)

Data are expressed as *n* (%) unless otherwise specified

*CRS* cytoreductive surgery, *HIPEC* hyperthermic intraperitoneal chemotherapy, *IQR* interquartile range (1st–3rd quartile), *BMI* body mass index, *ASA* American Society of Anesthesiologists, *CRC* colorectal cancer, *PM* peritoneal metastases, *PMP* pseudomyxoma peritonei, *LAMN* low-grade appendiceal mucinous neoplasm, *CT* computed tomography, *MRI* magnetic resonance imaging, *PET* positron emission tomography, *DLS* diagnostic laparoscopy

### Intraoperative Outcomes

The intraoperative outcomes including the anatomical resections are listed in Table 2. The median PCI was 5 (IQR 3–11), the median operation time was 468 min (IQR 368–599), and the median amount of intraoperative blood loss was 800 mL (IQR 350–2100). In all patients, a complete cytoreduction (i.e. CC-0 or CC-1) was achieved. During CRS, a median of four anatomical structures were resected, and the most common resections were the omentum (96.9%), small bowel (68.8%), rectum (56.3%), sigmoid (50.0%), and ovaries (28.1%). A stoma was created in 19 patients (59.4%).

**Table 2:** Treatment characteristics from all 32 patients who successfully underwent CRS + HIPEC

CRS + HIPEC procedure	
PCI at HIPEC (median [IQR])	5 [3–11]
Total anatomic resections (median [IQR])	4 [4–6]
Stomach	1 (3.1)
Jejunum	5 (15.6)
Ileum	9 (28.1)
Ileocecal	8 (25.0)
Appendix	4 (12.5)
Right colon	5 (15.6)
Transverse colon	1 (3.1)
Left colon	7 (21.9)
Sigmoid	16 (50.0)
Rectum	18 (56.3)
Omentum	31 (96.9)
Right diaphragm	5 (15.6)
Left diaphragm	3 (9.4)
Right peritoneum	6 (18.8)
Left peritoneum	5 (15.6)
Lymph nodes	5 (15.6)
Spleen	2 (6.3)
Gallbladder	6 (18.8)
Bladder	2 (6.3)
Urether	4 (12.5)
Uterus	5 (15.6)
Ovaries	9 (28.1)
Anastomoses	
0	11 (34.4)
1	12 (37.5)
≥ 2	9 (28.1)
Stoma post HIPEC	19 (59.4)
Operation time, min (median [IQR])	468 [368–599]
Blood loss, mL (median [IQR])	800 [350–2100]
Resection status	
CC-0	30 (93.8)
CC-1	2 (6.3)
*Postoperative recovery*	
Length of hospital stay, days (median [IQR])	17 [13–24]
Reoperation	7 (21.9)
In-hospital mortality	1 (3.1)
Complication rate, Clavien–Dindo grade	
I–II	10 (31.2)
III–IV	12 (37.5)
Complication type grade III or higher	
Anastomotic leakage	2 (6.3)
Postoperative bleeding	1 (3.1)
Intra-abdominal abscess	5 (15.6)
Wound infection	5 (15.6)
Wound dehiscence	3 (9.4)
Fistula formation	2 (6.3)
Urinoma	1 (3.1)
Electrolyte disorder	1 (3.1)

Data are expressed as *n* (%) unless otherwise specified

*CRS* cytoreductive surgery, *HIPEC* hyperthermic intraperitoneal chemotherapy, *IQR* interquartile range (1st–3rd quartile), *PCI* peritoneal cancer index, *CC* completeness of cytoreduction, *Clavien*–*Dindo* Clavien–Dindo classification system

### Overall Ability to Predict the Extent of Surgery (ES)

The number of resections necessary to achieve a complete cytoreduction were predicted correctly 24 times (18.3%), overestimated 57 times (43.5%), and underestimated 50 times (38.2%).

The overall PPV for the different anatomical structures ranged between 33.3 and 87.8% (Tables 3 and 4). The anatomical sites in which the surgeon predicted reasonably well were the appendix (100%), rectum (87.8%), and sigmoid (81.3%). On the other hand, to predict a resection of, in particular, the bladder (41.7%), jejunum (40.0%), peritoneum (34.2%), transverse colon (33.3%), or lymph nodes (32.1%) turned out to be very difficult. In contrast, the overall PPV for the presence of a stoma post-HIPEC was overall high (88.1%). In the 24 ES forms including the patients in whom a stoma was already present prior to CRS + HIPEC, a PPV of 100% was found.

**Table 3:** Overall positive and negative predictive values of the different gastrointestinal anatomical structures and the presence of a stoma post HIPEC

Anatomical structure	Surgeon’s prediction	Resected during CRS + HIPEC	*In situ* after CRS + HIPEC	PPV (%)	NPV (%)	Sensitivity (%)	Specificity (%)
Stomach	Resection	0	0	^a^	96.2	0.0	100
	Remains in situ	5	126				
Duodenum	Resection	0	0	^a^	100	^b^	100
	Remains in situ	0	131				
Jejunum	Resection	8	12	40.0	89.2	28.6	89.2
	Remains in situ	12	99				
Ileum	Resection	11	9	55.0	85.6	40.7	91.3
	Remains in situ	16	95				
Ileocecal	Resection	7	8	46.7	78.4	21.9	91.9
	Remains in situ	25	91				
Appendix	Resection	9	0	100	93.4	52.9	100
	Remains in situ	8	114				
Right colon	Resection	17	12	58.6	97.1	85.0	89.2
	Remains in situ	3	99				
Transverse colon	Resection	2	4	33.3	98.4	50.0	96.9
	Remains in situ	2	123				
Left colon	Resection	15	12	55.6	83.7	46.9	87.9
	Remains in situ	17	87				
Sigmoid	Resection	39	9	81.3	59.0	53.4	84.5
	Remains in situ	34	49				
Rectum	Resection	43	6	87.8	54.9	53.8	88.2
	Remains in situ	37	45				

	Surgeon’s prediction	Stoma after CRS + HIPEC	No stoma after CRS + HIPEC	PPV (%)	NPV (%)	Sensitivity (%)	Specificity (%)
Stoma post HIPEC	Yes	37	5	88.1	56.2	51.3	90.9
	No	39	50				

Data are expressed as *n*

*CRS* cytoreductive surgery, *HIPEC* hyperthermic intraperitoneal chemotherapy, *PPV* positive predictive value, *NPV* negative predictive value, *ES* extent of surgery

^a^In none of the 131 ES forms was removal of the anatomical structure predicted, therefore the overall PPV could not be calculated

^b^In none of the 131 ES forms was the anatomical structure resected during CRS + HIPEC, therefore the overall sensitivity could not be calculated

**Table 4:** Overall positive and negative predictive values for the other anatomical structures

Anatomical structure	Surgeon’s prediction	Resected during CRS + HIPEC	*In situ* after CRS + HIPEC	PPV (%)	NPV (%)	Sensitivity (%)	Specificity (%)
Right diaphragm	Resection	10	9	52.6	91.1	50.0	91.9
	Remains in situ	10	102				
Left diaphragm	Resection	8	6	57.1	95.7	61.5	94.9
	Remains in situ	5	112				
Right peritoneum	Resection	22	18	55.0	91.2	73.3	82.1
	Remains in situ	8	83				
Left peritoneum	Resection	25	48	34.2	98.3	96.2	45.7
	Remains in situ	1	57				
Lymph nodes	Resection	9	19	32.1	93.2	56.3	83.5
	Remains in situ	7	96				
Spleen	Resection	6	3	66.7	95.1	50.0	97.5
	Remains in situ	6	116				
Pancreas	Resection	0	0	^a^	100.0	^b^	100
	Remains in situ	0	131				
Gallbladder	Resection	4	3	57.1	85.5	18.2	97.2
	Remains in situ	18	106				
Bladder	Resection	5	7	41.7	96.6	55.6	94.3
	Remains in situ	4	115				
Ureter	Resection	6	3	66.7	94.3	46.1	97.5
	Remains in situ	7	115				
Uterus^c^	Resection	11	13	45.8	76.9	47.8	75.5
	Remains in situ	12	40				
	Remains in situ	7	23				

Data are expressed as *n*

*CRS* cytoreductive surgery, *HIPEC* hyperthermic intraperitoneal chemotherapy, *PPV* positive predictive value, *NPV* negative predictive value, *ES* extent of surgery

^a^In none of the 131 ES forms was removal of the anatomical structure predicted, therefore the overall PPV could not be calculated

^b^In none of the 131 ES forms was the anatomical structure resected during CRS + HIPEC, therefore the overall sensitivity could not be calculated

^c^Based on only 76 ES forms as these forms included information about female patients only

**Fig. 2:**
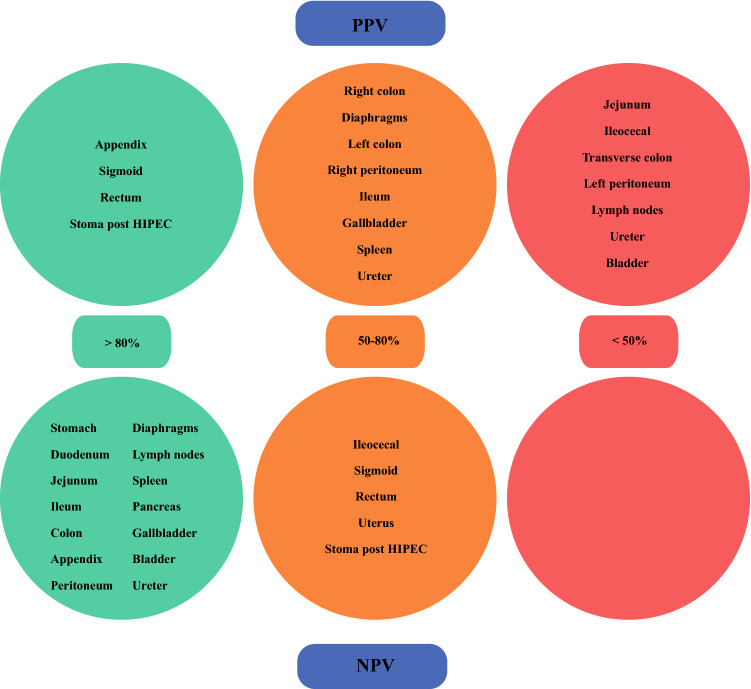
Overall positive and negative predictive values for all anatomical structures divided into good PPV/NPV (i.e. > 80%), moderate PPV/NPV (i.e. 50–80%), and poor PPV/NPV (i.e. < 50%). *PPV* positive predictive value, *NPV* negative predictive value, *HIPEC* hyperthermic intraperitoneal chemotherapy

The overall NPV for the different anatomical structures ranged between 54.9 and 100%, including 12 anatomical structures with an NPV > 90%. The lowest scores were found for the rectum (54.9%) and sigmoid (59.0%). In addition, the NPV for the absence of a stoma post-HIPEC was 56.2%. This means that surgeons incorrectly predicted the absence of a stoma in 56.2% of cases, as, in these patients, a stoma was created during CRS + HIPEC. In the 24 ES forms including patients in whom a stoma was already present prior to CRS + HIPEC, an NPV of 62.5% was found (Fig. 2).

The sensitivity for resection of the different gastrointestinal anatomical structures was overall low, with a range of 0.0–85.0%. In particular, overall sensitivity for the small bowel resections (i.e. jejunum, ileum, and ileocecal) was only 28.6%, 40.7%, and 21.9%, respectively. For the other anatomical structures, sensitivity was the highest for the left (96.2%) and right (73.3%) peritoneums. Overall sensitivity for the presence of a stoma post-HIPEC was found to be 51.3%.

The specificity for the preservation of the different anatomical structures was overall high and ranged from 75.5 to 100%.

### Individual Ability to Predict the ES

The individual ability to predict the ES is outlined in electronic Supplementary Tables 1 and 2. Major differences in PPVs between the surgeons were observed for most of the anatomical structures, with the exception of the appendix. For nine anatomical structures, the range between individual PPVs even exceeded 50% (i.e. jejunum, ileum, left colon, transverse colon, spleen, gallbladder, bladder, lymph nodes, and uterus). The largest differences were observed for the transverse colon (range 100%), uterus (range 87.5%), and ileum (range 83.3%).

In contrast, the NPVs for the majority of the anatomical structures were similar between surgeons and differences ranged between 1.5 and 31.8%. The largest difference was observed for the sigmoid (i.e. range 31.8%).

### Impact of Radiological Examinations on the Ability to Predict the ES

According to the preoperative evaluation for CRS + HIPEC, 87 ES forms (66.4%) included patients in whom only CT was performed and 44 ES forms (33.6%) included patients in whom both CT and MRI were performed. Combining CT with MRI significantly improved the overall PPV for the sigmoid (85.2 vs. 76.2%; *p* < 0.0001), rectum (100 vs. 73.9%; *p* < 0.0001), and ureter (100 vs. 40.0%; *p* = 0.024), and the overall NPV for the jejunum (100 vs. 85.4%; *p* = 0.001). For the other anatomical structures, no significant differences were found for the overall PPV and NPV.

### Postoperative Morbidity and Mortality

Major postoperative complications (i.e. grades III–IV) occurred in 12 patients (37.5%) (Table 2). The most common surgical complications in these patients were intra-abdominal abscesses (15.6%) and wound infections (15.6%). Reoperation was necessary in seven patients (21.9%). The overall 90-day postoperative mortality rate (i.e. grade V) was 3.1%.

## Discussion

In this prospective observational cohort study consisting of 131 ES forms, the surgeons’ ability to predict the ES prior to CRS + HIPEC was evaluated for the first time. Overall, the surgeons seemed to be able to better predict the anatomical structures that remain in situ after CRS + HIPEC, rather than predict the resections that were necessary to achieve a complete cytoreduction, with an underestimation of the ES in almost 40% of cases.

Over the past decades, CRS + HIPEC has improved survival outcomes for patients with PM from various primary tumors.^1^–^7^ This potential survival benefit needs to be in balance with the associated risks of treatment-related morbidity and mortality. HIPEC surgeons attempt to make this estimation for their patients in advance of planning a CRS + HIPEC procedure, but the complex interplay of patient, tumor, and treatment-related characteristics makes this task almost impossible. In recent years, various risk factors for the occurrence of major postoperative complications after CRS + HIPEC (i.e. grades III–V) have been identified.^17^,^19^,^20^,^26^ The ES, including the number of resected anatomical structures, has repeatedly been described as an independent risk factor for treatment-related morbidity.^19^,^20^,^26^ A surgeon’s ability to correctly predict the ES in advance of CRS + HIPEC seems to be one of the key elements to estimating the individual risk for treatment-related morbidity, which is of importance for informing patients in the outpatient clinic and for patient-shared decision making. In our current study, we show that despite the presence of different state-of-the-art imaging modalities such as multidetector CT, PET imaging, and MRI, surgeons predicted the number of resections correctly in only 18.3% of cases. Furthermore, in 38.2% of cases, an underestimation of the number of anatomical resections occurred; subsequently, the associated risk for treatment-related morbidity might also be underestimated prior to surgery. The high PPV for the presence of a stoma (88.1%) and the low NPV for the absence of a stoma (56.2%) post-HIPEC supports our protocol with stoma counseling and education for every patient prior to CRS + HIPEC.

To our knowledge, no previous studies have reported on a surgeon’s ability to predict the ES prior to CRS + HIPEC. However, the large number of publications about the limitations of current imaging modalities in detecting PM and the occurrence of an open–close procedure in up to 50% of patients, confirm that, in most patients, despite having performed extensive imaging, surgeons will only discover the true extent of peritoneal disease during the exploratory laparotomy itself.^27^–^33^ This has major logistical consequences as, for instance, an open-close procedure is not only a patient tragedy but also a drawback for the other patients waiting on the list. This is also reflected by our current study showing both an overestimation and underestimation of the number of resected anatomical structures during CRS + HIPEC in 43.5 and 38.2% of cases, respectively.

Interestingly, for some specific anatomical structures, we found high PPVs for each of the individual surgeons. For the appendix, this might be explained by the clear indication to remove this organ during CRS + HIPEC standardly. The PPVs for the sigmoid and rectum were also high, which might be explained by the relatively high number of patients with synchronous onset of colorectal PM from sigmoid or rectal cancer (25.0 and 18.8%, respectively), and where the surgeon knows in advance that this part of the colon will have to be removed. Overall, NPVs for the different anatomical structures were higher and showed less variation between surgeons compared with the PPVs. This suggests that surgeons were better able to predict the anatomical structures that remained in situ after CRS + HIPEC than to predict the anatomical structures that would be resected during CRS + HIPEC to achieve a complete cytoreduction.

From a clinical perspective, PPVs and NPVs are the most interesting outcomes reflecting the ability to predict the ES according to our daily practice. The ES that seems to be necessary to achieve a complete cytoreduction plays a crucial role in the selection process for CRS + HIPEC. This estimation per anatomical structure in advance is expressed by the PPV and NPV. However, our study results should be interpreted with some caution as, from a statistical point of view, it is known that both values are influenced by the prevalence of the performed resections during CRS + HIPEC. For example, in our study cohort, there is a relatively high number of patients with rectal cancer, making it easier for surgeons to predict a rectum resection, resulting in a higher PPV. On the other hand, it is more difficult to predict the preservation of the rectum, resulting in a lower NPV. In summary, the PPVs and NPVs provide specific information about the ability of our surgeons to predict the ES in this specific study population.

This study has certain strengths and limitations. This is the first study that describes the ability of experienced surgeons to predict the ES prior to CRS + HIPEC. These results have been collected in a way that fully reflects our daily clinical practice by having only experienced surgeons complete the standardized ES forms prior to surgery for a group of patients who represent our average HIPEC population. Gathered knowledge from this study made us aware of the still-existing challenge of predicting the ES, and future research should focus on optimizing the detection of PM during the preoperative work-up. There is not just one CRS + HIPEC procedure; these procedures are very different in extent and burden, and thus outcomes, and a better estimation of the ES prior to surgery will improve our preoperative decision making, especially when we are dealing with patients who are older or have extensive comorbidity. Our study has some limitations due to the single-center approach and the already-mentioned limitations of the PPV and NPV from a statistical point of view. Our surgeons are extensively trained to perform gastrointestinal procedures and CRS + HIPEC procedures and therefore these results might not be extrapolated to all centers, although most CRS + HIPEC procedures are performed in highly experienced centers these days. The limitations of the PPV and NPV have been partly overcome by also presenting the sensitivity and specificity for the different anatomical structures as these outcomes are not influenced by the number of resections. However, sensitivity and specificity seem less useful for surgeons because they do not reflect daily practice as the predictions for the ES are always made prior to surgery.

## Conclusions

The ES during CRS + HIPEC is a well-known risk factor for the occurrence of major postoperative morbidity, and is therefore essential to know prior to surgery. Surgeons with extensive experience in performing these procedures have the ability to predict in advance which of the anatomical structures can be preserved during CRS + HIPEC but in most cases fail to predict the actual ES, including the resections that are necessary to achieve a complete cytoreduction. This phenomenon has not been described before and emphasizes that future research should focus even more on optimizing the detection of the extent of disease prior to surgery.

### Electronic supplementary material

Below is the link to the electronic supplementary material.

Supplementary material 1 (DOCX 18 kb)

Supplementary material 2 (DOCX 18 kb)
