# Supplementary material for: Surgeons’ Ability to Predict the Extent of Surgery Prior to Cytoreductive Surgery with Hyperthermic Intraperitoneal Chemotherapy
Source: Ann Surg Oncol. 2020 Feb 12;27(8):2997–3008. doi: 10.1245/s10434-020-08237-8 (PMC7334271; doi:10.1245/s10434-020-08237-8)
Supplement: Supplementary file 1 — Supplementary material 1 (DOCX 18 kb) [file 10434_2020_8237_MOESM1_ESM.docx]

**Supplementary Table 1 – Positive and negative predictive values for all anatomical structures divided per surgeon.**

| **Anatomical structure** | **Surgeon I**  *N = 21* | | **Surgeon II**  *N = 28* | | **Surgeon III**  *N = 30* | | **Surgeon IV**  *N = 30* | | **Surgeon V**  *N = 22* | |
| --- | --- | --- | --- | --- | --- | --- | --- | --- | --- | --- |
|  | **PPV (%)** | **NPV (%)** | **PPV (%)** | **NPV (%)** | **PPV (%)** | **NPV (%)** | **PPV (%)** | **NPV (%)** | **PPV (%)** | **NPV (%)** |
| **Stomach** | ***** | 95.2 | ***** | 96.4 | ***** | 96.7 | ***** | 96.7 | ***** | 95.5 |
| **Duodenum** | ***** | 100.0 | ***** | 100.0 | ***** | 100.0 | ***** | 100.0 | ***** | 100.0 |
| **Jejunum** | ***** | 90.5 | 60.0 | 91.3 | 28.6 | 87.0 | 28.6 | 82.6 | 100.0 | 95.2 |
| **Ileum** | 0.0 | 84.2 | 83.3 | 90.9 | 25.0 | 80.8 | 60.0 | 80.0 | 66.7 | 94.7 |
| **Ileocecal** | ***** | 66.7 | ***** | 82.1 | 60.0 | 80.0 | 33.3 | 74.1 | 42.9 | 93.3 |
| **Appendix** | ***** | 90.5 | 100.0 | 92.3 | 100.0 | 96.4 | 100.0 | 96.4 | 100.0 | 89.5 |
| **Right colon** | 75.0 | 100.0 | 60.0 | 100.0 | 75.0 | 92.3 | 44.4 | 100.0 | 57.1 | 93.3 |
| **Transverse colon** | ***** | 95.0 | 0.0 | 100.0 | 0.0 | 96.6 | 100.0 | 100.0 | 50.0 | 100.0 |
| **Left colon** | 100.0 | 94.1 | 66.7 | 84.0 | 60.0 | 80.0 | 27.3 | 84.2 | 75.0 | 77.8 |
| **Sigmoid** | 87.5 | 53.8 | 75.0 | 56.3 | 75.0 | 50.0 | 88.9 | 60.0 | 81.8 | 81.8 |
| **Rectum** | 100.0 | 50.0 | 83.3 | 50.0 | 80.0 | 50.0 | 100.0 | 40.0 | 75.0 | 64.3 |
| **Right diaphragm** | 66.7 | 88.9 | 40.0 | 95.7 | 33.3 | 88.9 | 66.7 | 88.9 | 60.0 | 94.1 |
| **Left diaphragm** | 66.7 | 100.0 | 75.0 | 100.0 | ***** | 92.9 | 50.0 | 96.2 | 100.0 | 90.5 |
| **Right peritoneum** | 62.5 | 100.0 | 44.4 | 84.2 | 50.0 | 90.0 | 62.5 | 90.9 | 60.0 | 94.1 |
| **Left peritoneum** | 33.3 | 91.7 | 50.0 | 100.0 | 46.2 | 100.0 | 27.3 | 100.0 | 36.4 | 100.0 |
| **Lymph nodes** | 75.0 | 100.0 | 80.0 | 100.0 | 20.0 | 90.0 | ***** | 81.8 | 0.0 | 95.2 |
| **Spleen** | 100.0 | 100.0 | 100.0 | 96.3 | ***** | 90.0 | 25.0 | 96.2 | 100.0 | 95.0 |
| **Pancreas** | ***** | 100.0 | ***** | 100.0 | ***** | 100.0 | ***** | 100.0 | ***** | 100.0 |
| **Gallbladder** | 100.0 | 90.0 | 100.0 | 92.6 | 40.0 | 88.0 | ***** | 80.0 | ***** | 77.3 |
| **Bladder** | ***** | 95.2 | ***** | 92.9 | 100.0 | 100.0 | 22.2 | 100.0 | 100.0 | 95.2 |
| **Ureter** | 50.0 | 94.7 | 100.0 | 96.3 | 66.7 | 96.3 | 66.7 | 92.6 | ***** | 90.9 |
| **Uterus** | 40.0 | 75.0 | 60.0 | 81.8 | 100.0 | 80.0 | 12.5 | 60.0 | 75.0 | 87.5 |
| **Stoma post HIPEC** | 100.0 | 53.8 | 90.0 | 55.6 | 87.5 | 54.5 | 66.7 | 50.0 | 90.0 | 75.0 |

***** In none of the ES forms of the surgeon removal of the anatomical structure was predicted and therefore the PPV could not be calculated.

**PPV** positive predictive value, **NPV** negative predictive value
