# Supplementary material for: Surgeons’ Ability to Predict the Extent of Surgery Prior to Cytoreductive Surgery with Hyperthermic Intraperitoneal Chemotherapy
Source: Ann Surg Oncol. 2020 Feb 12;27(8):2997–3008. doi: 10.1245/s10434-020-08237-8 (PMC7334271; doi:10.1245/s10434-020-08237-8)
Supplement: Supplementary file 2 — Supplementary material 2 (DOCX 18 kb) [file 10434_2020_8237_MOESM2_ESM.docx]

| **Anatomical structure** | **Surgeon I**  *N = 21* | | **Surgeon II**  *N = 28* | | **Surgeon III**  *N = 30* | | **Surgeon IV**  *N = 30* | | **Surgeon V**  *N = 22* | |
| --- | --- | --- | --- | --- | --- | --- | --- | --- | --- | --- |
|  | **Sensitivity (%)** | **Specificity (%)** | **Sensitivity (%)** | **Specificity (%)** | **Sensitivity (%)** | **Specificity (%)** | **Sensitivity (%)** | **Specificity (%)** | **Sensitivity (%)** | **Specificity (%)** |
| **Stomach** | 0.0 | 95.2 | 0.0 | 100 | 0.0 | 100 | 0.0 | 100 | 0.0 | 100 |
| **Duodenum** | **¥** | 100 | **¥** | 100 | **¥** | 100 | **¥** | 100 | **¥** | 100 |
| **Jejunum** | 0.0 | 100 | 60.0 | 91.3 | 40.0 | 80.0 | 33.3 | 79.2 | 50.0 | 100 |
| **Ileum** | 0.0 | 88.9 | 71.4 | 95.2 | 16.7 | 87.5 | 37.5 | 90.9 | 66.7 | 94.7 |
| **Ileocecal** | 0.0 | 100 | 0.0 | 100 | 37.5 | 90.9 | 12.5 | 90.9 | 75.0 | 77.8 |
| **Appendix** | 0.0 | 100 | 50.0 | 100 | 66.7 | 100 | 66.7 | 100 | 60.0 | 100 |
| **Right colon** | 100 | 94.4 | 100 | 92.0 | 60.0 | 96.0 | 100 | 80.8 | 80.0 | 82.4 |
| **Transverse colon** | 0.0 | 95.0 | **¥** | 100 | 0.0 | 96.6 | 100 | 100 | 100 | 95.2 |
| **Left colon** | 80.0 | 100 | 33.3 | 95.5 | 37.5 | 90.9 | 50.0 | 66.7 | 42.9 | 93.3 |
| **Sigmoid** | 53.8 | 87.5 | 56.3 | 75.0 | 35.2 | 84.6 | 50.0 | 92.9 | 81.8 | 81.8 |
| **Rectum** | 60.0 | 100 | 55.6 | 80.0 | 44.4 | 83.3 | 55.6 | 100 | 54.5 | 81.8 |
| **Right diaphragm** | 50.0 | 94.1 | 66.7 | 88.0 | 25.0 | 92.3 | 40.0 | 96.0 | 75.0 | 88.9 |
| **Left diaphragm** | 100 | 94.7 | 100 | 96.0 | 92.9 | 0.0 | 66.7 | 92.6 | 33.3 | 100 |
| **Right peritoneum** | 100 | 81.3 | 57.1 | 76.2 | 71.4 | 78.3 | 62.5 | 87.0 | 75.0 | 88.9 |
| **Left peritoneum** | 75.0 | 64.7 | 100 | 72.7 | 100 | 45.8 | 100 | 33.3 | 100 | 61.1 |
| **Lymph nodes** | 100 | 94.4 | 100 | 95.8 | 50.0 | 69.2 | 0.0 | 75.0 | 0.0 | 95.2 |
| **Spleen** | 100 | 100 | 50.0 | 100 | 0.0 | 100 | 50.0 | 89.3 | 66.7 | 100 |
| **Pancreas** | **¥** | 100 | **¥** | 100 | **¥** | 100 | **¥** | 100 | **¥** | 100 |
| **Gallbladder** | 33.3 | 100 | 33.3 | 100 | 40.0 | 88.0 | 0.0 | 100 | 0.0 | 100 |
| **Bladder** | 0.0 | 100 | 0.0 | 100 | 100 | 100 | 100 | 75.0 | 50.0 | 100 |
| **Ureter** | 50.0 | 94.7 | 50.0 | 100 | 66.7 | 96.3 | 50.0 | 96.2 | 0.0 | 100 |
| **Uterus** | 50.0 | 66.7 | 60.0 | 81.8 | 40.0 | 100 | 20.0 | 46.2 | 75.0 | 87.5 |
| **Stoma post HIPEC** | 57.1 | 100 | 52.9 | 90.9 | 41.2 | 92.3 | 25.0 | 85.7 | 75.0 | 90.0 |

**Supplementary Table 2 – Sensitivity and specificity for all anatomical structures divided per surgeon.**

**¥** In none of the ES forms from this surgeon this anatomical structure was resected during CRS+HIPEC, and therefore sensitivity could not be calculated.
